# Plants: Sources of Diversity in Propolis Properties

**DOI:** 10.3390/plants11172298

**Published:** 2022-09-02

**Authors:** Otilia Bobiş

**Affiliations:** Department of Apiculture and Sericiculture, Faculty of Animal Science and Biotechnology, University of Agricultural Sciences and Veterinary Medicine Cluj-Napoca, 400372 Cluj-Napoca, Romania; obobis@usamvcluj.ro

**Keywords:** propolis, plants, chemical composition, bioactive properties

## Abstract

Propolis or “bee glue” is a resinous waxy substance that is produced by honeybees (*Apis mellifera)* by mixing the exudates collected from plants, namely tree buds, sap flows, leaves, branches and barks with their saliva and beeswax. Propolis composition is very complex. Its main constituents are resins and volatiles originating from plants and wax added by the bee. The biological activity of propolis is assigned to these plant-derived substances. The main three types of propolis are European propolis, called poplar type propolis; Green Brazilian propolis (derived mainly from the leaf resin of *Baccharis dracunculifolia*) and Red Cuban propolis (from the floral resin of *Clusia rosea*). The plant’s source gives it a specific composition and properties for the propolis types that are coming from different regions of the world. For this reason, studies on the chemical composition of propolis as well as its botanical sources resulting in its geographically conditioned diversity, were a very good theme for the present Special Issue (SI) of *Plants* journal. The present SI contains nine original contributions addressing propolis plant sources, their chemical composition and different bioactive properties derived from this origin. The chemical composition of propolis that is made by the bees was also discussed, as well as the different medical activities of propolis extract. The papers cover a wide range of subjects, including (i) the plant species used by the bees as raw material for propolis production, (ii) the biological activities of plant extracts related to propolis, (iii) the chemical composition of different types of propolis, (iv) the biological activity of propolis, (v) propolis and human health, and (vi) synergism between plants and propolis in human health. The studies have been carried out in both in vitro and in vivo surveys and a wide range of geographic regions are covered in the sample collection.

## 1. Introduction

The aim of this Special Issue, entitled “Plants: Sources of Diversity in Propolis Properties”, was proposed to expand our knowledge about propolis, a natural product having as raw materials different substances from plants, which honeybees (*Apis mellifera)* collect and transform in order to protect their hive. This Special Issue contains nine scientific articles (six original research articles and three original reviews), contributing to a better knowledge of this valuable bee product that may lead to the development of new drugs for the prevention and treatment of numerous diseases. This Editorial was written to summarize the valuable papers that are published in this Special Issue (SI) and to lead to a wider visibility and citation of these studies. Propolis composition depends on several factors. The classification of propolis is based on its geographical location, color and agricultural characteristics. It is also classified according to the flora where the bees collect the resins, which represent the raw material for propolis production.

## 2. Overview of SI

Kurek-Górecka et al. (2022) [1] published a research paper entitled “Comparison of the Antioxidant Activity of Propolis Samples from Different Geographical Regions”. The paper describes different propolis samples originating from Turkey, Poland, Romania and Uruguay. Propolis possesses high antioxidant activity, determined by its phenolic compounds. Due to diverse composition and possible impact on human health, eight samples of propolis were evaluated for their phenolic composition and antioxidant activity. Samples of Polish, Romanian, Turkish and Uruguayan origin propolis were used for a phenolic spectrum determination using high performance liquid chromatography and photodiode array detection, and in vitro DPPH and ABTS methods were used to determine the antioxidant activity of the extracts. Different statistical (PCA and HCA) models were applied to evaluate the correlation between the isolated polyphenols and the antioxidant activity. The results confirmed that there is variability in propolis composition depending on the geographical region of collection and the plant sources, and there is a correlation between the chemical composition and the antioxidant activity. The results of the PCA and HCA analyses confirmed that Polish propolis is similar to that from different provinces of Romania, while Turkish and Uruguay are completely different. Polish and Romanian propolis belong to the poplar type. The assessed phenolic compounds of propolis samples that were used in the study are responsible for its antioxidant effect. The observed antioxidant activity of the analyzed samples may suggest directing subsequent research on the prophylactic and therapeutic properties concerning cardiovascular, metabolic, neurodegenerative, and cancerous diseases, which are worth investigating.

Another published study investigated the antimicrobial effects of a mouthwash containing propolis and the effect of a propolis paste formulation on dental healing after tooth extraction in patients with periodontal disease [2]. In the mouthwash study, the population comprised 40 patients, which were divided as follows: the control mouthwash, 0.2% chlorhexidine (*v*/*v*) mouthwash, 2% (*w*/*v*) propolis mouthwash, and propolis + chlorhexidine mouthwash. The study of the propolis paste comprised a population of 60 patients with periodontal disease, and a total of 120 symmetric tooth extractions were performed. Propolis showed antimicrobial activity by itself, and especially with the chlorhexidine association. Three days after the surgery, in the teeth treated with control paste, only 13.4% had completely healed; however, with the propolis paste, in 90% of the periodontal sockets, the healing was complete. In addition, a reduction in *Streptococci mutans* and *Lactobacilli* cfu was observed with propolis, and especially with the association of chlorhexidine + propolis. Propolis mouthwash reduced bacterial proliferation, especially in association with chlorhexidine. Propolis paste is a viable alternative for socket healing after dental extraction. The knowledge gained from these findings will provide a foundation for similar propolis therapies in order to improve the healing process after dental surgery. 

Alvear et al. (2021) [3] described how the geographical area of the collection determines the chemical composition and antimicrobial potential of Chilean propolis. The biological properties of Chilean propolis have been described in many publications over time, and these include antibacterial, antifungal and antibiofilm activities. Clinical experiences with synthetic antibiotics indicated the need to discover new sources of bioactive compounds that are associated with ethnopharmacological knowledge or natural sources, such as propolis. The microscopic analysis of pollen grains from plants allows us to determine the botanical origin of the propolis samples. In different regions of Chile (Angol, Maule, and Melipilla), presented different, region specific pollen grains (*Sorghum bicolor*; *Lotus* sp.; *Acacia* sp.; *Pinus radiata*, *Eucalyptus* sp.; *Salix babylonica*; *Quillaja saponaria* or other species). Colorimetric assays were performed to quantify the total number of polyphenols present in Chilean propolis samples established that the sample collected from Angol region showed high amounts of phenolics compounds, with significant statistical differences in comparison with the other samples. The main compounds that were identified were pinocembrin, quercetin and caffeic acid phenethyl ester (CAPE). The Angol sample showed a high content of polyphenols. Studies that determine the influence of geographical and floral variables on the chemical composition of propolis are a valuable source of information for the study of its biological properties. 

Lithuanian propolis was described by Stanciauskaite et al. (2021) [4]. Balsam poplar and black poplar (*Populus balsamifera* L. and *Populus nigra* L.) buds that grow in Lithuania are the primary source of propolis, therefore it is proper to evaluate and compare the composition of these raw plant materials and propolis, quantitatively and qualitatively. Propolis and balsamic poplar bud extract are dominated by *p*-coumaric acid and black poplar-caffeic acid. The antioxidant activity was evaluated by DPPH (2,2-diphenyl-1-picrylhydrazyl), ABTS (2,2-azino-bis (3-ethylbenzothiazoline-6-sulfonic acid), FRAP (ferric-reducing antioxidant power) and CUPRAC (cupric reducing antioxidant capacity) methods and all extracts showed antioxidant activity, and obtained results that correlated with the obtained amounts of phenolic compounds and flavonoids in the extracts. Studies of antimicrobial activity have shown that all extracts have a growth inhibitory effect against *Staphylococcus aureus* and *Candida albicans*, but the extract of balsam poplar buds showed the most significant effect of such kind. Considering the results of the research, it can be stated that balsam poplar buds that are cultured in Lithuania are the primary raw material of propolis, which is rich in phenolic compounds with antioxidant properties and is a promising raw material for pharmaceutical purposes. 

Pain is one of the most common symptoms encountered in the medical practice. New treatment strategies for pain management are needed. In this respect, propolis has been used in traditional medicine to relieve various types of pain. The aim of the study that was published by Al-Hariri and Abualait (2020) [5] was to investigate the potential effects of the green Brazilian propolis alcohol extract in vivo on the nociceptive and inflammatory pain models in rats. Rats were distributed into three random groups (*n* = 6); Group I: control group received the normal saline, intraperitoneally (i.p.); Group II: treated with green Brazilian propolis alcohol extract (P50 mg/kg i.p.); Group III: treated with P100 mg/kg i.p. After sixty minutes, 50 μL of 5% formalin was injected subcutaneously into the dorsal surface of the right hind paw. The nociceptive response was identified by counting the number of flinches of the injected paw. The number of flinches was counted for the period of 0–5 min (early phase; neurogenic) and 10–60 min (late phase; inflammatory). Thermal hyperalgesia was assessed using a three-paw withdrawal latency measurement with ten minutes intervals, using a planter analgesic meter. Abdominal writhe (contraction) was induced by the i.p. injection of acetic acid (1 mL of 2%). The results showed that green Brazilian propolis alcohol extract caused a significant inhibition of acetic acid-induced pain and significantly increased the pain threshold against infrared and formalin tests. The promising antinociceptive and anti-inflammatory properties of propolis and/or its active constituents as natural compounds in the present study indicates that it merits further studies in pain. 

The modern techniques used in propolis research are described in the study performed by Seven et al. (2020) [6], to determine the effects of chitosan-coated nano-propolis (NP), which is synthesized via a green sonochemical method, and propolis on the side effects of cisplatin (CP), which is a widely used drug in the treatment of cancer. For this aim, 56 rats were divided into seven groups, balancing their body weights (BW). The study was designed as such: control, CP (3 mg/kg BW at single dose of CP as intraperitoneal, ip), propolis (100 mg/kg BW per day of propolis by gavage), NP-10 (10 mg/kg BW of NP per day by gavage), CP + propolis (3 mg/kg BW of CP and 100 mg/kg BW of propolis), CP + NP-10 (3 mg/kg CP and 10 mg/kg BW of NP), and CP + NP-30 (3 mg/kg BW of CP and 30 mg/kg BW of NP). Propolis and NP (especially NP-30) were preserved via biochemical parameters, oxidative stress, and the activation of apoptotic pathways (anti-apoptotic protein: Bcl-2, and pro-apoptotic protein: Bax) in liver and kidney tissues in the toxicity that was induced by CP. The NP were more effective than propolis at a dose of 30 mg/kg BW and had the potential to ameliorate CP’s negative effects while overcoming serious side effects, such as liver and kidney damage. 

The studies that are published as research articles in this SI will be followed by others, due to the fact that propolis research is a wide area of study and this natural product may be used to design a multitude of medicinal products to fight against different bacterial or viral diseases.

The review studies published in the present Special Issue describe both propolis properties and the role of plant sources in the determination of propolis properties. 

Propolis is a resinous mixture, made by the honeybees from substances collected from tree or other plant buds, plant exudates, or resins found in the stem, branches, or leaves of different plants. The geographical origin of propolis is given by the plant sources from their respective areas. Balica et al. (2021) [7], document the potential role of propolis in the prevention and treatment of metabolic disorders. In the last few decades, propolis has been extensively researched, with multiple studies confirming its anti-inflammatory, antioxidant, antimicrobial, and wound-healing properties. More recently, due to an exponential increase in the number of patients with metabolic diseases, there is also a growing interest in the study of the antidiabetic, antihyperlipidemic, and anti-obesity effects of propolis. The aim of this review was to evaluate the potential role of propolis in the prevention and treatment of metabolic diseases like diabetes mellitus, dyslipidemia, and obesity. The preclinical in vivo and in vitro pharmacological models investigating antidiabetic, antihyperlipidemic, and anti-obesity effects of propolis were reviewed with a focus on the putative mechanisms of the actions of several chemical constituents. Additionally, the available clinical studies and an evaluation of the safety profile of propolis were also presented. Different studies have classified this bee product according to the vegetal material from the same areas. Poplar-type propolis has the widest spread in the world, in the temperate zones from Europe, Asia, and North America. The name is given by the main plant source from where the bees are collecting the resins, although other vegetal sources are present in the mentioned areas. Different *Pinus* spp., *Prunus* spp., *Acacia* spp. and also *Betula pendula*, *Aesculus hippocastanum*, and *Salix alba* are important sources of resins for “poplar-type” propolis. The aim of the review published by Dezmirean et al. (2021) [8] was to identify the vegetal material’s chemical composition and the activities of plant resins and balms used by the bees to produce poplar-type propolis and to compare it with the final product from similar geographical regions. The relevance of this review is to find the similarities between the chemical composition and properties of plant sources and propolis. The latest determination methods of bioactive compounds from plants and propolis are also reviewed. Moise and Bobiş (2020) [9] have reviewed the main plant sources of green and red propolis and their implications in their bioactive properties. Nowadays, propolis is used as a highly valuable product in alternative medicine for improving health or treating a large spectrum of pathologies, as an ingredient in pharmaceutical products, and as a food additive. Different vegetal materials are collected by honeybees and mixed with wax and other own substances in order to obtain the final product, called propolis. It is known as the bee product with the widest chemical composition due to the raw materials that are collected by the bees. Different types are known worldwide: green Brazilian propolis (having *Baccharis dracunculifolia* as the major plant source), red Brazilian propolis (from *Dalbergia ecastophyllum*), European propolis (*Populus nigra* L.), Russian propolis (*Betula verrucosa Ehrh*), Cuban and Venezuelan red propolis (*Clusia* spp.), etc. An impressive number of scientific papers have already demonstrate the pharmacological potential of different types of propolis, the most important of these activities being the antimicrobial, anti-inflammatory, antitumor, immunomodulatory, and antioxidant activities. However, the bioactive compounds that are responsible for each activity have not been fully elucidated. This review collected important data about the chemical composition and bioactive properties of the vegetal sources and compared them with the chemical composition of respective propolis types, in order to determine the connection between the floral source and the propolis properties.

## 3. Conclusions

The research studies that are contained in this SI describe both chemical composition of propolis and the bioactive properties, including antioxidant, antibacterial, and antinociceptive activities. Propolis extracts (mainly hydro-alcoholic) are demonstrated to have good bioactive properties, being used in different medical sectors. It is demonstrated that the biological activities of propolis are derived from the secondary metabolites of plants, namely phenolics, which the bees collect from the resins and plant materials collected from the different geographical locations. In this way, the link between plants and final product propolis is demonstrated. The review articles that are published in our SI are of great importance, one of them [8] being classified as highly cited paper in Web of Science, after only several months from publication. We are glad that all our published papers have different numbers of citations, showing the quality of the studies and also the interest given by readers and scientists for the respective studies.
